# Mobile-Based Platform With a Low-Calorie Dietary Intervention Involving Prepackaged Food for Weight Loss for People With Overweight and Obesity in China: Half-Year Follow-Up Results of a Randomized Controlled Trial

**DOI:** 10.2196/47104

**Published:** 2024-10-28

**Authors:** Xi Wang, Suyuan Wang, Lingyu Zhong, Chenghui Zhang, Yanhong Guo, Mingxia Li, Li Zhao, Shuming Ji, Junjie Pan, Yunhong Wu

**Affiliations:** 1 Department of Endocrinology, Hospital of Chengdu Office of People’s Government of Tibetan Autonomous Region, Chengdu, China; 2 Tibet Autonomous Region Clinical Research Center for High-Altitude Stress Endocrinology and Metabolism Disease, Hospital of Chengdu Office of People’s Government of Tibetan Autonomous Region, Chengdu, China; 3 Department of Clinical Nutrition, Hospital of Chengdu Office of People’s Government of Tibetan Autonomous Region, Chengdu, China; 4 Department of Health Policy and Management, West China School of Public Health and West China Fourth Hospital, Sichuan University, Chengdu, China; 5 Department of Clinical Research Management, West China Hospital of Sichuan University, Sichuan University, Chengdu, China; 6 Department of Cardivascular Medicine, Huashan Hospital Affilicated to Fudan University, Shanghai, China

**Keywords:** weight loss, obesity, body fat, food replacement product, meal replacement, weight, obese, RCT, randomized, mHealth, mobile health, mobile app, mobile application, mobile phone

## Abstract

**Background:**

Obesity is a rapidly increasing health problem in China, causing massive economic and health losses annually. Many techniques have emerged to help people with obesity better adhere to intervention programs and achieve their weight loss goals, including food replacement and internet-delivered weight loss consultations. Most studies on weight loss interventions mainly focused on the change in body weight or BMI; however, body fat, especially visceral fat mass, is considered the main pathogenic factor in obesity. In China, more reliable evidence is required on this topic. Moreover, it is unclear whether an integrated weight loss program combining food replacement products, mobile app-based platforms, and daily body composition monitoring using a wireless scale is useful and practical in China.

**Objective:**

In this 2-arm, parallel-designed, randomized study, we explored the effectiveness and safety of the Metawell (Weijian Technologies Inc) weight loss program in China, which combines prepackaged biscuits, a wireless scale, and a mobile app.

**Methods:**

Participants in the intervention group were guided to use food replacement products and a scale for weight loss and monitoring, whereas participants in the control group received printed material with a sample diet and face-to-face education on weight loss at enrollment. The intervention lasted for 3 months, and follow-up visits were conducted at months 3 and 6 after enrollment. Dual-energy x-ray absorptiometry and quantitative computed tomography were used to assess body fat. A multilevel model for repeated measurements was used to compare differences between the 2 groups.

**Results:**

In total, 220 patients were randomly assigned to intervention (n=110) and control (n=110) groups. Participants in the intervention group had significantly greater decreases in BMI, total body fat, visceral adipose area, and subcutaneous adipose area (all *P*<.001) than those in the control group. However, the rate of change in lean mass was not significantly different between the 2 groups (*P*=.62). Further, 35 participants in the intervention group reported adverse events. Constipation was the most frequently reported adverse event (11/110), followed by dizziness (6/110), hypoglycemia (4/110), fatigue (3/110), and gastritis (3/35).

**Conclusions:**

The Metawell program was effective for weight loss. After the intervention, participants in the intervention group lost more body weight and body fat while retaining muscle mass than those in the control group.

## Introduction

In China, the prevalence of obesity has increased rapidly from 3.1% in 2004 to 8.1% in 2018 [1]. Obesity is closely related to a series of diseases such as cardiovascular diseases, diabetes, and hypertension and leads to physical impairments and a decrease in the quality of life [2]. The economic losses caused by overweight and obesity in China are estimated to be US $12.97 billion [3]. Moreover, an elevated BMI was estimated to be the seventh highest risk factor for death in China, equating to nearly 0.7 million deaths in 2017 [4].

Nutrition therapy is considered to be the foundation of obesity management [5]. Meal replacement products provide an easy way for participants to follow a weight loss program, and rapid weight loss during the initial stage of the intervention can help to build the confidence of participants, thus leading to greater weight loss. The Doctor Referral of Overweight People to Low Energy Total Diet Replacement Treatment (DROPLET) study revealed that total meal replacement programs provided by general practitioners are effective for weight loss [6], and a meta-analysis showed that partial or total meal replacements were associated with greater weight loss [7,8].

The delivery of lifestyle intervention programs may also have an impact on their effectiveness in weight loss. Whether the use of the internet or a mobile app platform to deliver the intervention will lead to greater weight loss is controversial. A meta-analysis showed that the use of mobile apps can improve the effect of weight loss interventions [9], whereas other studies did not find a statistically significant effect for mobile apps [10].

Most studies of weight loss interventions focused on the change in body weight or BMI; however, body fat, especially visceral fat mass, is considered the main pathogenic factor in obesity [11]. We found 1 randomized study focused on the change in body composition during a weight loss intervention in China [12]. However, the sample size was limited, and the body composition results are not sufficiently accurate considering their measuring method; therefore, we still need more reliable evidence on this topic. Furthermore, it is unknown whether an integrated weight loss program combining food replacement products, a mobile app-based platform, and daily monitoring of body composition using a wireless scale would be useful and practical in China. Thus, we performed this study to evaluate the safety and efficacy of the Metawell program, a weight loss program using prepackaged biscuits, a Bluetooth body composition scale, and a mobile app for uploading body composition data and delivering meal guidance from health care professionals daily for weight loss and changes in body composition and body fat distribution.

## Methods

### Study Design

This was a single-center, open-label, 2-arm, parallel-designed, randomized controlled trial; all participants were recruited from Chengdu City via social media platforms and face-to-face visits, and assessments will take place at enrollment and at 3 and 6 months after enrollment. The trial started on April 11, 2019. Participants were randomly assigned to the intervention and control groups at a ratio of 1:1. This trial was registered in the Chinese Clinical Trial Registry (ChiCTR1900021630). After our trial started, several participants older than 65 years showed a strong willingness to participate; after discussion, we decided to raise the upper limit of the participant aged from 65 to 75 years.

### Ethical Considerations

This trial was approved by the ethics committee of the Hospital of Chengdu Office of People’s Government of the Tibetan Autonomous Region (2019-No.01). All participants have signed the informed consent; all data are anonymized. All participants will be informed that they will receive ¥200 (a currency exchange rate of ¥6.92=US $1 is applicable) compensation for each follow-up visit they attend.

### Eligibility Criteria

The inclusion criteria were those aged between 18 and 75 years with a BMI ≥25 and <40 kg/m^2^ and one or more of the following: a history of hypertension or either systolic blood pressure >120 mm Hg or diastolic blood pressure >80 mm Hg, abdominal circumference >96 cm (90 cm for women), fasting triglyceride level >1.69 mmol/L, a history of type 2 diabetes mellitus managed with lifestyle (not on insulin or oral medications) or fasting blood glucose level >5.6 mmol/L, and high-density lipoprotein cholesterol level <1.04 mmol/L (1.3 mmol/L for women).

Participants with one or more of the following conditions were excluded: a history of coronary artery disease; diabetes mellitus managed with insulin or any oral hypoglycemic pill for diabetes; glucose intolerance or fasting glucose level ≥8 mmol/L; congestive heart failure; familial hypercholesterolemia, including familial hypertriglyceridemia, fasting low-density lipoprotein cholesterol level >4.2 mmol/L, fasting triglyceride level >6.8 mmol/L, and current use of lipid-lowering agents; a past medical history of hypothyroidism, Cushing syndrome, eating disorders, gout in the past 6 months, confirmed episodes of hypoglycemia, pregnancy, advanced liver disease, renal insufficiency, or any other major chronic medical condition; and smokers who planned to quit smoking in the following 12 months. Participants with hypertension were included only if they were taking fewer than 3 antihypertensive medications, did not have changes in the dose of their blood pressure medications in the last month, and had systolic blood pressure <160 mm Hg and diastolic blood pressure <100 mm Hg. Finally, people who could not use smartphones were excluded.

### Randomization, Masking, and Blinding

The list of random numbers was generated using R with a certain random seed, the results of which were sequentially sealed in opaque envelopes and were unsealed while participants signed the consent and finished eligibility screening. This study was an open-label study; patients and researchers were not blinded.

### Interventions

Details of the interventions had been described in our previously published protocol [13]. In general, participants in the intervention group would download a mobile app and be guided to use food replacement products and a scale for weight loss and monitoring. Their diet, body composition, and urine ketone will be monitored every day, and trained instructors will guide them remotely. During the weight loss stage (0‐3 months), participants will be guided to take food replacement biscuits along with a selection of healthy recipes (such as seaweed soup, skimmed milk, spiced beef, grains, vegetables) and advised to take multivitamin tablets daily. Detailed nutritional information on diet replacement biscuits is listed in Table S1 in Multimedia Appendix 1, and the overall intake of energy for the intervention group was restricted to 800‐1200 kcal/day; the instructor will guide participants to adjust their recipe based on their health condition and the speed of weight loss. During the weight maintenance stage (3‐6 months), participants will stop using the biscuit, and they will be told to monitor their weight and upload data. When noticeable weight regain is detected, the practitioner will initiate a 2‐ to 3-day weight loss intervention using the same protocol as that used in the weight loss stage to maintain the participant’s body weight. Based on obesity management guidelines, a calorie intake of 800‐1200 is generally safe. Furthermore, on analyzing the data of over 250,000 Chinese using this program for weight loss, we found no significant safety concerns.

Meanwhile, participants in the control group would receive printed material with a sample diet, as well as face-to-face education on weight loss at enrollment. The printed sample diet used for the control group included a daily energy intake of 1500 kcal for men and 1200 kcal for women. Doctors and the dietician in our research group (XW, L Zhong, and YW) were responsible for monitoring participants’ safety.

### Outcomes

The primary outcome was weight loss as a result of the intervention, defined as the reduction in BMI after 6 months. The following two secondary outcomes were also analyzed: (1) the proportion of participants achieving a body weight reduction of greater or equal to 10% of baseline body weight and (2) the change in body fat mass and lean mass, as well as changes in the distribution of adipose tissue.

### Assessment and Follow-Up

Participants were followed up at 3 and 6 months after enrollment. During each follow-up assessment, body weight was measured using an ultrasonic height-weight scale (DHM-200, Dinghengkeji), and physical activity was assessed using the International Physical Activity Questionnaire long form. Dual-energy x-ray absorptiometry (DXA) was used to assess the body composition of the participants using a GE Lunar Prodigy scanner (GE Healthcare). A 16-slice computed tomography (CT) scanner (Aquilion 16; TOSHIBA) was used to perform abdominal scanning, and the distribution of adipose tissue was analyzed with QCTPRO software (Mindways Software) using a slice between L2 and L3. DXA and CT were performed only at baseline and at the 6-month follow-up.

### Sample Size

According to the DROPLET study [6], we hypothesized that the average absolute difference in weight loss between the trial and control groups would be at least 4 kg in weight, with an SD of 9 kg. Therefore, 100 participants per group will provide more than 90% power to detect this difference, with an α error of .05. The sample size would be 110 participants per group considering an estimated 10% attrition rate.

### Statistical Analysis

The intention-to-treat dataset was used to analyze the primary and secondary outcomes. The normality of data was tested using Kolmogorov-Smirnov test. All of our continuous data had nonnormality according to test results, so they are presented as median (IQR). Categorical data are presented as count (%). Baseline information was compared between the 2 groups, Mann-Whitney *U* test and chi-square test were used for continuous and categorical variables, respectively. A multilevel model for repeated measurements was used to test the interaction of the intervention and time against BMI as the primary outcome and fat mass, lean mass, visceral adipose area, and subcutaneous adipose area as the secondary outcomes. Variants of different individuals were treated as the first level and different follow-up times were treated as the second level. After reviewing previous studies such as he DROPLET study and incorporating with clinical experience of clinicians in our group, baseline body weight, physical activity, sex, and age were adjusted. The rate of missing data was relatively low in this study; therefore, missing data were not considered in the analysis.

We performed a post hoc subgroup analysis to determine whether there were any differences among participants of different age and sex groups. Baseline characteristics were compared using SPSS (version 24.0; IBM Corp), and multilevel model analysis was performed using MLwiN (version 2.30; Centre for Multilevel Modelling, University of Bristol). *P* values <.05 were considered statistically significant.

## Results

### Overview

**Figure 1.**
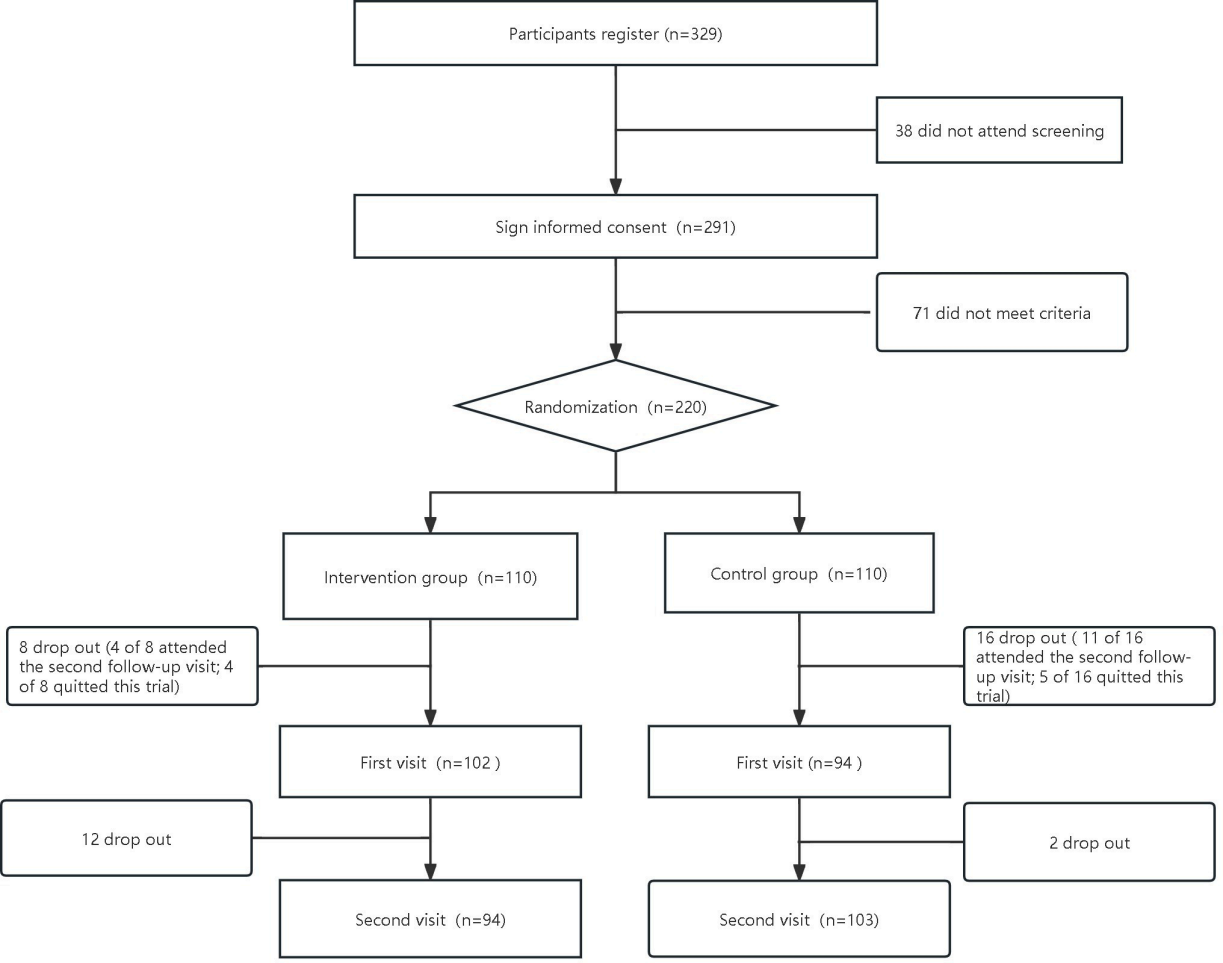
Flowchart of this study.

Participants were recruited between April 2019 and January 2020. A total of 329 participants were registered for screening and 220 were randomly assigned to the intervention and control groups (n=110 each; Figure 1). The median age of the participants was 33 (IQR 28-41) years and 57.8% (n=125) were women. The median BMI was 27.9 (IQR 4) kg/m^2^. There were no differences in any of the baseline characteristics between the 2 groups (Table 1). At the 6-month follow-up, we followed 103 participants in the intervention group and 94 in the control group (Figure 1).

**Table 1.** Baseline information of the participants.

		Overall (n=220)	Intervention group (n=110)	Control group (n=110)	*P* value
**Sex, n (%)**					.63
	Male	95 (42.22)	48 (44.86)	47 (41.59)	
	Female	125 (57.78)	59 (55.14)	66 (58.41)	
Age (years), median (IQR)		33 (28-41)	32 (28- 39)	33 (28-41)	.48
Height (cm), median (IQR)		164.5 (158.0-171.0)	165.0 (159.0-170.5)	164 (158.0-171.0)	.85
Weight (kg), median (IQR)		76.4 (68.9-86.1)	77.4 (70.1-85.3)	75.2 (67.8-86.2)	.49
BMI (kg/m^2^), median (IQR)		27.9 (26.2-29.9)	28.2 (26.3-30.9)	27.7 (26.0-29.7)	.39
Waist circumference (cm), median (IQR)		97.4 (92.4-102.8)	97.7 (93.0-102.5)	97.1 (92.1-102.9)	.57
Hip circumference (cm), median (IQR)		105.4 (101.3-110.0)	105.5 (100.2-109.8)	106.4 (102.1-110.0)	.18
Subcutaneous adipose (cm^2^), median (IQR)		201.5(158.7-271.1)	208.8 (167.5-275.7)	196.9 (155.1-267.8)	.33
Visceral adipose (cm^2^), median (IQR)		167.9 (123.2-230.7)	171.6 (127.4-215.4)	163.3 (120.5-237.1)	.54
Total fat mass (kg), median (IQR)		29.4 (25.1-34.9)	29.8 (25.6-34.7)	29.35 (24.6-35.6)	.96
Total lean mass (kg), median (IQR)		46.1 (39.1-55.0)	47.5 (39.1-54.9)	43.83 (39.1-55.1)	.67
Physical activity (METs^a^/week), median (IQR)		1062.5 (258.0-2715.0)	1284 (346.5-2806.0)	928.5 (102.5-2331.0)	.33

a MET: metabolic equivalent of task.

### Primary Outcome

At 6 months, 31 (28%) and 3 (3%) participants in the intervention and control groups, respectively, achieved a weight loss of at least 10% compared with their baseline weight. The median change in BMI was –1.90 (IQR –0.8 to –3.2) kg/m^2^ for the intervention group and –0.03 (IQR –0.9 to –0.4) kg/m^2^ for the control group (Table 2).

**Table 2.** Primary and secondary outcomes by group.

Outcomes	Intervention group	Control group
**3 months**		
BMI change (kg/m^2^), median (IQR)	–2.3 (–3.2 to –1.3)	–0.2 (–0.9 to 0.4)
At least 10% weight lost, n (%)	37 (33.63)	5 (4.54)
**6 months**		
BMI change (kg/m^2^), median (IQR)	–1.9 (–0.8 to –3.2)	–0.03 (–0.9 to 0.4)
At least 10% weight lost, n (%)	31 (28.18)	3 (2.72)
Body fat mass change (kg), median (IQR)	–3.9 (–7.3 to –1.3)	0.1 (–1.9 to 2.6)
Body lean mass change (kg), median (IQR)	–0.8 (–2.1 to 0.2)	–0.4 (–1.7 to 0.6)
Subcutaneous adipose change (cm^2^), median (IQR)	–37.6 (–71.4 to –10.4)	–11.2 (–34.9 to 18.8)
Visceral adipose change (cm^2^), median (IQR)	–15.1 (-44.3 to 2.4)	7.8 (–12.6 to 37.9)

**Figure 2.**
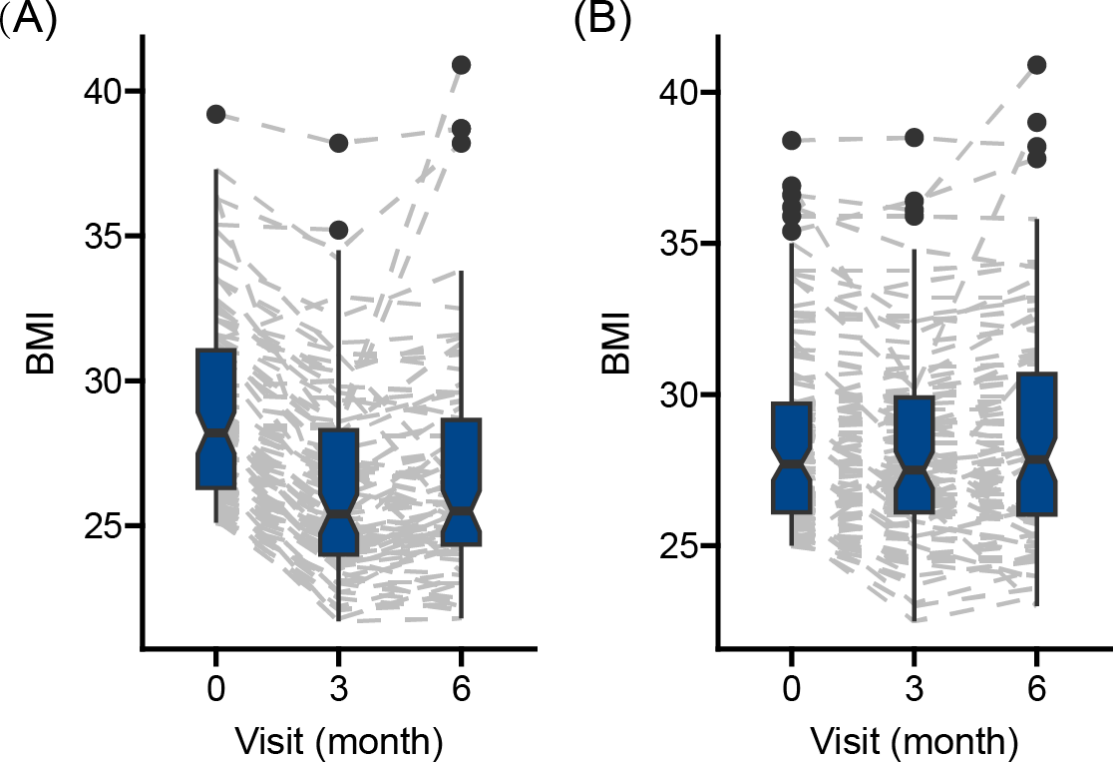
BMI change over 6 months to (A) intervention group and (B) control group.

The change in BMI was different between the 2 groups before (β_group×time_=–1.214, *P*<.001) and after (β_group×time_=–1.238, *P*<.001) adjustment for sex, age, baseline body weight, and baseline physical activity level (Figure 2; Table S2 in Multimedia Appendix 2).

### Secondary Outcomes

**Figure 3.**
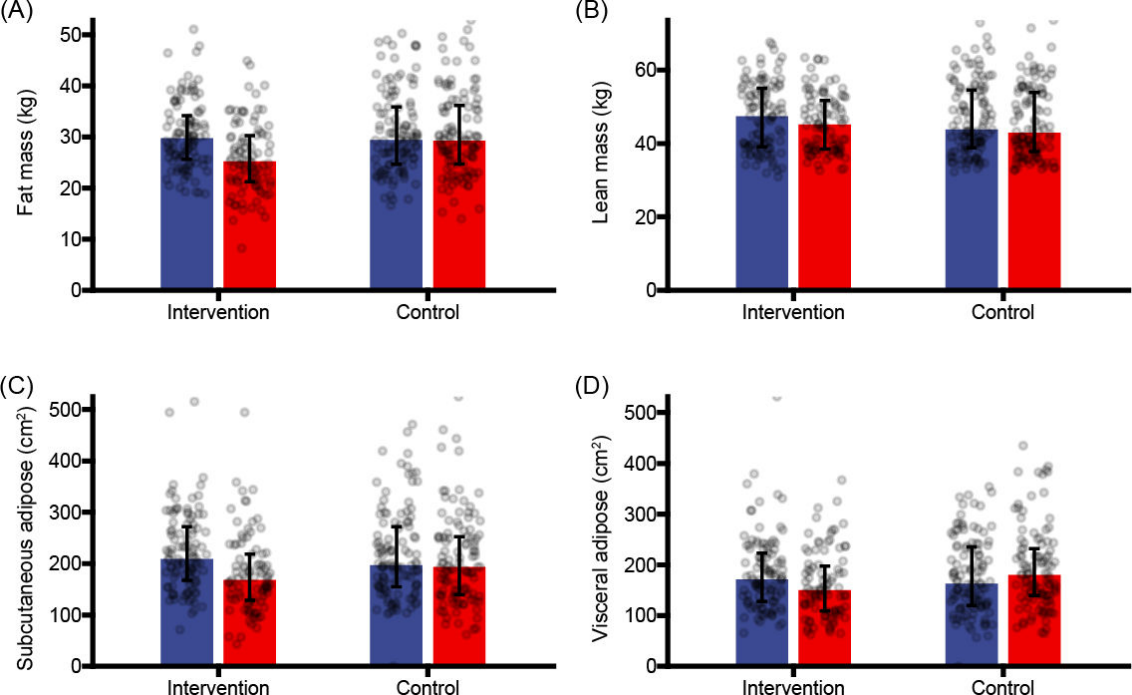
Change in body composition before (blue) and after (red) the intervention in different groups: (A) total body fat mass, (B) total lean mass, (C) subcutaneous adipose area, and (D) visceral adipose area.

After the intervention, the total body fat mass decreased by 5.07 kg and the total body lean mass decreased by 3.13 kg in the intervention group. In the control group, body fat mass increased by 0.06 kg, and body lean mass decreased by 0.27 kg (Figure 3). The change rate of body fat mass between the 2 groups was statistically significant (β_group×time_=–4.127, *P*<.001), whereas that of total muscle mass was not significant (β_group×time_=–0.177, *P*=.62; Table S2 in the Multimedia Appendix 2).

Subcutaneous and visceral adipose areas decreased by 53 and 31 cm^2^, respectively, in the intervention group (Figure 3). In the control group, subcutaneous adipose area decreased by 1 cm^2^ and visceral adipose area increased by 19 cm^2^. The rates of change in subcutaneous adipose area (β_group×time_=–40.898, *P*<.001) and visceral adipose area (β_group×time_=–40.409, *P*<.001) were different between the 2 groups (Table S3 in Multimedia Appendix 3).

### Subgroup Analysis

Subgroup analyses were performed to determine whether the intervention was effective in the different sex and age groups. The decrease rate in BMI was greater in the intervention group in both male and female participants (β_group×time_=–1.781 for male sex and β_group×time_=–0.833 for female sex; Table S4 in Multimedia Appendix 4). Furthermore, for participants in all age groups, the rate of decrease in BMI was greater in the intervention group (β_group×time_=–0.754 for age ≤30 years, β_group×time_=–0.833 for age 31‐40 years, and β_group×time_=–2.055 for age ≥41 years; Table S4 in Multimedia Appendix 4).

### Adverse Events

In the control group, only 2 participants reported 4 adverse events (AEs) during the follow-up visits, whereas 35 participants reported AEs in the intervention group (Table 3). The most common AE in the intervention group was constipation (11/110); 10% of the participants in the intervention group reported varying degrees of constipation during the follow-up visits. Other common AEs were dizziness (6/35), hypoglycemia (4/110), fatigue (3/110), and gastritis (3/110). All AEs were classified as mild to moderate, none of these participants were hospitalized because of AEs.

**Table 3.** Numbers of participants reporting adverse events.

	Intervention group, n	Control group, n	Total, n
Constipation	11	1	12
Hypoglycemia	4	0	4
Diarrhea	1	1	2
Fatigue	3	1	4
Dizziness	6	1	7
Sour regurgitation	1	0	1
Hair loss	1	0	1
Flustered	1	0	1
Rhinitis	2	0	2
Gastritis	3	0	3
Menstrual disorder	1	0	1
Elevated urinary ketones	1	0	1

## Discussion

### Principal Results

This investigation demonstrated that the implementation of a weight loss program combining food replacement products, remote guidance by practitioners, and daily monitoring of body composition is effective for weight loss and reducing fat mass, especially visceral fat, without significant muscle loss in people with overweight and obesity in China.

### Comparison With Prior Work

According to previous investigations, more than half of patients with obesity were not motivated to adhere to weight loss interventions; the most frequently reported barrier was a lack of accessibility to weight loss diets and a lack of support and external control [14]. Furthermore, the need for intensive follow-up with motivational interviews was highlighted. In our program, a series of methods were used to address these barriers, including using food replacement products, intensive guidance delivered via a mobile app, and a wireless scale provided to participants for their daily monitoring of body composition. Food replacement products can be a useful tool in weight loss programs, providing an easy way for participants to adhere to the diet intervention [14]. Mobile apps enable frequent interactions between health care professionals and patients and allow intensive motivational interviews. Additionally, more frequent body composition monitoring is closely associated with better adherence. Several studies have revealed that the use of food replacement products, mobile apps, and wireless scales can lead to greater weight loss [7,15-17]. Our study confirms that the combination of these techniques can lead to greater weight loss than traditional educational materials.

Although BMI is a good marker for obesity screening, the threshold varies among different ethnic and age groups. Moreover, considering that most comorbidities of obesity are mediated by excess fat, endocrine and immune responses of adipose tissue are distributed throughout different organs [18,19]. Hence, BMI is not suitable to reflect the complexity of obesity. In recent years, some studies on weight loss interventions have investigated the change in body composition and adipose tissue distribution; however, most used bioelectrical impedance analysis for the measurements, which is less accurate than DXA or CT [20]. In our study, we used DXA and quantitative CT for a more accurate assessment of body composition changes, and we confirmed that the intervention program could reduce the amount of total fat, visceral fat, and subcutaneous fat compared with the control program. Therefore, we can be more confident that patients with obesity can achieve health benefits through the loss of body fat by adhering to this intervention program.

We also found that the change rate in lean mass was not statistically significant (*P*=.62) between the 2 groups. In contrast, previous studies suggested that severe calorie-restricting interventions would lead to a greater loss of lean mass [21]. The use of a food replacement product might be a reason why the participants in the intervention group did not experience a significant loss of lean mass, despite having a restricted intake of calories. A systematic review by Coleman et al [22] indicated that food replacement products can minimize the loss of lean mass by reducing the variety of foods. Furthermore, our intervention provided a relatively high protein intake level while restricting calorie intake. Increasing the daily intake of protein can prevent muscle loss while decreasing the amount of body fat [23].

The safety of food replacement products is a concern for health care professionals, and their use is not recommended by most guidelines because of the lack of data on safety and effectiveness. Our study demonstrated that, although the risk was slightly higher in the intervention group, the side effects of this product were mild to moderate. The most common AE was constipation, which is consistent with a previous study on total food replacement products and low-carbohydrate diets [6]. To assess the safety of our intervention program, we also analyzed the change in spinal bone mineral density (BMD), we found the BMD decreased by about 2% (from mean 1.11, SD 0.10 g/cm^2^ to mean 1.09, SD 0.09 g/cm^2^, *P*<.01) in the intervention group (data not shown). However, considering that the least significant change of our DXA measurement was 5.3%, which is much higher than the decreasing rate we found in our study, we believe that this change was not clinically significant. Therefore, food replacement programs are relatively safe for weight loss in healthy individuals.

The follow-up of some participants was delayed owing to the COVID-19 pandemic and the lockdown in Chengdu City; however, considering that our participants did not have access to our weight loss intervention during the lockdown and according to relevant studies [24,25], we expected their body weight to increase, resulting in the underestimation of the effect size; therefore, we did not treat this situation.

### Limitations and Strengths

To the best of our knowledge, this is the first study using DXA and quantitative CT to accurately and systematically evaluate change in body fat, visceral adipose tissue, and subcutaneous adipose tissue during weight loss intervention, which is more relative to participants’ health than BMI [26,27]. This is the main highlight of our study.

The main limitation of our study is that the follow-up period was relatively short to observe the long-term effects of this intervention. Meanwhile, we found a statistically significant (although without clinical significance) decrease in BMD in the intervention group, it remains unclear whether there would be any lasting impact on BMD after the intervention ended. Therefore, an extended follow-up will be performed to investigate whether participants will regain their body weight and whether their BMD will continue to decrease. Furthermore, we only explored the effectiveness of this weight loss program without performing a cost-effectiveness analysis. We will collect and analyze related data in the following study. Moreover, this study’s results should be treated as exploratory outcomes, and further studies are required to verify them.

### Conclusions

The Metawell program was effective for weight loss. After the intervention, participants in the intervention group lost more body weight and body fat while retaining muscle mass than those in the control group.

## Supplementary material

10.2196/47104 Multimedia Appendix 1 Nutritional information of Yufit biscuit.

10.2196/47104 Multimedia Appendix 2 The effect of the intervention on the change in BMI.

10.2196/47104 Multimedia Appendix 3 The effect of the intervention on body composition changes.

10.2196/47104 Multimedia Appendix 4 Results of the subgroup analysis.

10.2196/47104 Checklist 1 CONSORT (Consolidated Standards for Reporting Trials) checklist.

## Abbreviations

AE: adverse event
BMD: bone mineral density
CT: computed tomography
DROPLET: Doctor Referral of Overweight People to Low Energy Total Diet Replacement Treatment
DXA: dual-energy x-ray absorptiometry
