# Supplementary material for: Mobile-Based Platform With a Low-Calorie Dietary Intervention Involving Prepackaged Food for Weight Loss for People With Overweight and Obesity in China: Half-Year Follow-Up Results of a Randomized Controlled Trial
Source: JMIR Mhealth Uhealth. 2024 Oct 28;12:e47104. doi: 10.2196/47104 (PMC11534272; doi:10.2196/47104)
Supplement: Multimedia Appendix 1 [file mhealth-v12-e47104-s001.docx]

**Table S1.** Nutritional information of Yufit biscuit.

| **Ingredients:**  Wheat Flour, Soybean Protein Flour, Soybean Oil, Eggs, Powdered Sugar, Lentinula Edodes, Black Fungus, Hericium Erinaceus, Grifola Frondosa, Bamboo Fungus, Seaweed, Cassia, Kumb, Emblica, Bran, Starch, Salt, Retinyl Acetate (Vitamin A), Thiamine Hydrochloride (Vitamin B1), Riboflavin (Vitamin B2), Pyridoxine Hydrochloride (Vitamin B6), Niacin, Folic Acid, Ferrous Sulfate, Calcium Carbonate, Zinc Gluconate, Sodium Selenite. | **Nutrition Facts** | | |
| --- | --- | --- | --- |
|  | Items | Per 100g | NRV% |
|  | Energy | 1792kJ | 21% |
|  | Protein | 10.2g | 17% |
|  | Fat | 16.1g | 27% |
|  | -Trans Fat | 0g |  |
|  | Carbohydrate | 53.4g | 18% |
|  | Dietary Fiber | 14.4g | 58% |
|  | Sodium | 315mg | 16% |
|  | Vitamin A | 13mgRE | 2% |
|  | Vitamin B1 | 0.46mg | 33% |
|  | Vitamin B2 | 0.88mg | 63% |
|  | Vitamin B6 | 0.43mg | 31% |
|  | Folic Acid | 105mgDFE | 26% |
|  | Calcium | 300mg | 38% |
|  | Iron | 4.6mg | 31% |
|  | Zinc | 1.61mg | 11% |
