# Supplementary material for: Mobile-Based Platform With a Low-Calorie Dietary Intervention Involving Prepackaged Food for Weight Loss for People With Overweight and Obesity in China: Half-Year Follow-Up Results of a Randomized Controlled Trial
Source: JMIR Mhealth Uhealth. 2024 Oct 28;12:e47104. doi: 10.2196/47104 (PMC11534272; doi:10.2196/47104)
Supplement: Multimedia Appendix 2 [file mhealth-v12-e47104-s002.docx]

**Table S2.** The effect of the intervention on the change in BMI.

|  | Model 1 |  |  | Model 2^a^ |  |
| --- | --- | --- | --- | --- | --- |
|  | β | p |  | Β | p |
| BMI change | -1.214 | <0.001 |  | -1.238 | <0.001 |

a. adjusted for sex, age, baseline body weight, and baseline physical activity.

BMI, body mass index
