# Supplementary material for: Mobile-Based Platform With a Low-Calorie Dietary Intervention Involving Prepackaged Food for Weight Loss for People With Overweight and Obesity in China: Half-Year Follow-Up Results of a Randomized Controlled Trial
Source: JMIR Mhealth Uhealth. 2024 Oct 28;12:e47104. doi: 10.2196/47104 (PMC11534272; doi:10.2196/47104)
Supplement: Multimedia Appendix 3 [file mhealth-v12-e47104-s003.docx]

**Table S3.** The effect of the intervention on body composition changes.

|  | β | P |
| --- | --- | --- |
| Total fat mass | -4.127 | <0.001 |
| Total lean mass | -0.177 | 0.622 |
| Subcutaneous adipose | -40.898 | <0.001 |
| Visceral adipose | -40.409 | <0.001 |
