# Supplementary material for: Mobile-Based Platform With a Low-Calorie Dietary Intervention Involving Prepackaged Food for Weight Loss for People With Overweight and Obesity in China: Half-Year Follow-Up Results of a Randomized Controlled Trial
Source: JMIR Mhealth Uhealth. 2024 Oct 28;12:e47104. doi: 10.2196/47104 (PMC11534272; doi:10.2196/47104)
Supplement: Multimedia Appendix 4 [file mhealth-v12-e47104-s004.docx]

**Table S4.** Results of the subgroup analysis.

|  | β | p |
| --- | --- | --- |
| Sex |  |  |
| Male | −1.781 | <0.001 |
| Female | −0.833 | <0.001 |
| Age |  |  |
| ≤30 | −0.754 | 0.006 |
| 31-40 | −1.021 | <0.001 |
| ≥40 | −2.055 | 0.007 |
